# 4′-Amino-2,2′′-dioxo-2,2′′,3,3′′-tetra­hydro-1*H*-indole-3-spiro-1′-cyclo­pent-3′-ene-2′-spiro-3′′-1*H*-indole-3′,5′,5′-tricarbonitrile dihydrate

**DOI:** 10.1107/S1600536808002146

**Published:** 2008-01-23

**Authors:** D. Gayathri, D. Velmurugan, G. Shanthi, P. T. Perumal, K. Ravikumar

**Affiliations:** a Centre of Advanced Study in Crystallography and Biophysics, University of Madras, Guindy Campus, Chennai 600 025, India; b Organic Chemistry Division, Central Leather Research Institute, Adyar, Chennai 600 020, India; c Laboratory of X-ray Crystallography, Indian Institute of Chemical Technology, Hyderabad 500 007, India

## Abstract

In the title compound, C_22_H_12_N_6_O_2_·2H_2_O, the cyclo­pentene ring adopts an envelope conformation, with the spiro C atom bonded to the dicyano-substituted C atom deviating by 0.437 (2) Å from the plane of the remaining four atoms in the ring. The puckering and smallest displacement asymmetry parameters for the ring are *q*_2_ = 0.275 (2) Å, ϕ = 212.4 (4)° and Δ_s_(C_2_) = 2.7 (2). The dihedral angle between the two indole groups is 60.1 (1)°. The structure contains inter­molecular N—H⋯O hydrogen bonds involving the indole groups and O—H⋯O and O—H⋯N hydrogen bonds involving the water mol­ecules.

## Related literature

For related literature, see: Akai *et al.* (2004 ▶); Cremer & Pople (1975 ▶); Gallagher *et al.* (1985 ▶); Nagata *et al.* (2001 ▶); Nardelli (1983 ▶); Williams & Cox (2003 ▶); Zaveri *et al.* (2004 ▶).
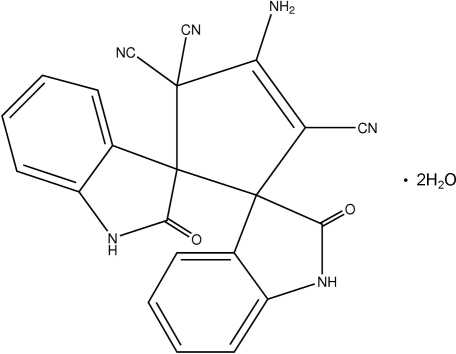


## Experimental

### 

#### Crystal data

C_22_H_12_N_6_O_2_·2H_2_O
*M* *_r_* = 428.41
Orthorhombic, 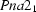

*a* = 17.1850 (16) Å
*b* = 8.9849 (9) Å
*c* = 13.3275 (13) Å
*V* = 2057.8 (3) Å^3^
*Z* = 4
Mo *K*α radiation
μ = 0.10 mm^−1^
*T* = 293 (2) K
0.25 × 0.24 × 0.20 mm

#### Data collection

Bruker SMART APEX CCD diffractometer
Absorption correction: none
16968 measured reflections
2551 independent reflections
2367 reflections with *I* > 2σ(*I*)
*R*_int_ = 0.022

#### Refinement

*R*[*F* ^2^ > 2σ(*F* ^2^)] = 0.042
*wR*(*F* ^2^) = 0.108
*S* = 1.06
2551 reflections
313 parameters
9 restraints
H atoms treated by a mixture of independent and constrained refinement
Δρ_max_ = 0.27 e Å^−3^
Δρ_min_ = −0.14 e Å^−3^

### 

Data collection: *SMART* (Bruker, 2001 ▶); cell refinement: *SAINT* (Bruker, 2001 ▶); data reduction: *SAINT*; program(s) used to solve structure: *SHELXS97* (Sheldrick, 2008 ▶); program(s) used to refine structure: *SHELXL97* (Sheldrick, 2008 ▶); molecular graphics: *PLATON* (Spek, 2003 ▶); software used to prepare material for publication: *SHELXL97* and *PARST* (Nardelli, 1995 ▶).

## Supplementary Material

Crystal structure: contains datablocks I, global. DOI: 10.1107/S1600536808002146/bi2273sup1.cif

Structure factors: contains datablocks I. DOI: 10.1107/S1600536808002146/bi2273Isup2.hkl

Additional supplementary materials:  crystallographic information; 3D view; checkCIF report

## Figures and Tables

**Table 1:** Hydrogen-bond geometry (Å, °)

*D*—H⋯*A*	*D*—H	H⋯*A*	*D*⋯*A*	*D*—H⋯*A*
N1—H1⋯O2^i^	0.86	2.09	2.905 (2)	158
N2—H2⋯O3^i^	0.86	2.22	3.032 (3)	157
O3—H3*A*⋯O1^ii^	0.85 (1)	2.00 (2)	2.795 (3)	157 (4)
O3—H3*B*⋯N6^iii^	0.85 (1)	2.22 (5)	2.834 (3)	129 (5)
O3—H3*B*⋯O4^iv^	0.85 (1)	2.62 (3)	3.381 (4)	149 (5)
O4—H4*A*⋯N4^v^	0.85 (1)	2.38 (3)	3.171 (5)	155 (7)
O4—H4*B*⋯N3	0.85 (1)	2.68 (4)	3.392 (4)	142 (6)
N5—H5*A*⋯O3^vi^	0.90 (1)	1.97 (1)	2.860 (3)	175 (3)
N5—H5*B*⋯O4^vi^	0.90 (1)	2.17 (1)	3.061 (4)	171 (3)

Symmetry codes: (i) 
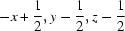
; (ii) 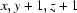; (iii) 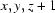; (iv) 
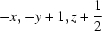
; (v) 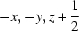; (vi) 
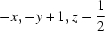
.

**Fig. 1.**
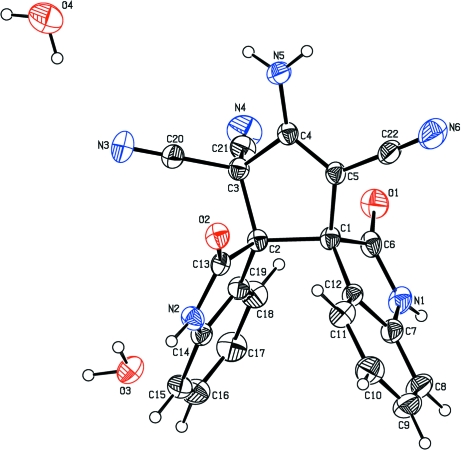
The molecular structure of title compound, showing 30% probability displacement ellipsoids for non-H atoms.

**Fig. 2.**
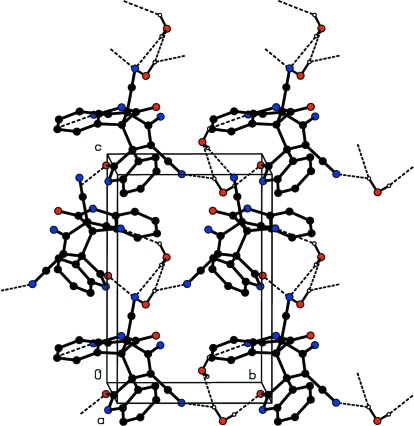
The molecular packing viewed down the a axis, showing O—H···N and O—H···O intermolecular interactions.

**Fig. 3.**
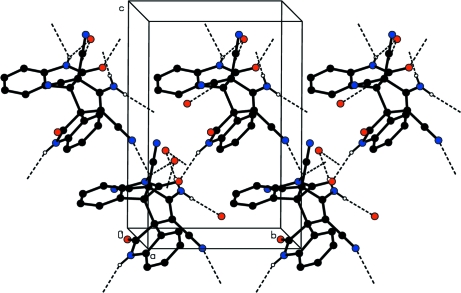
The molecular packing viewed down the a axis, showing N—H···O intermolecular interactions.

## supplementary crystallographic information

### Comment

The indole template is generally recognized as an important structure in medicinal chemistry, and in particular, oxindoles are important constituents of natural indole alkaloids as well as drugs in development and also in the clinic (Akai *et al.*, 2004). Among them, the spiro-oxindole framework is an important structural motif in biologically relevant compounds such as natural products and pharmaceuticals (Williams & Cox, 2003). The oxindole motif is present in the anti-Parkinson's drug ropinirole (Gallagher *et al.*, 1985), in non-opioid nociceptin receptor ligands (Zaveri *et al.*, 2004), and in the growth hormone secretagogues (Nagata *et al.*, 2001). As the title compound is biologically important, we have determined its crystal structure using X-ray diffraction.

One of the oxindole moieties (C2/C13/N2/C14/C19) is planar with atom O2 deviating by 0.127 (1) Å from the plane of five-membered ring. The dihedral angle between the five- (C2/C13/N2/C14/C19) and six-membered (C14—C19) rings is 1.6 (1)°. The cyclopentene ring adopts an envelope conformation with C2 deviating by 0.437 (2) Å from the plane of the rest of the atoms in the ring. The five-membered ring (N1/C6/C1/C12/C7) in the oxindole moieties adopts a slightly twisted conformation. The puckering parameters (Cremer & Pople, 1975) and the smallest displacement asymmetry parameters (Nardelli, 1983) for the cyclopentene ring and the five-membered ring (N1/C6/C1/C12/C7) are q_2_ = 0.275 (2)Å and 0.091 (2) Å, φ = 212.4 (4)° and 56.3 (12)° and Δ_s_(C_2_) = 2.7 (2) and Δ_2_(C_7_) = 0.5 (2), respectively.

### Experimental

To a stirred mixture of isatylidene malononitrile (2 mmol) and Hantzsch dihydropyridine ester (1 mmol) in ethanol (10 ml), a catalytic amount of indium(III) chloride (20 mol%) was added and the mixture was stirred at room temperature for about 1–2 h. After complete conversion (as indicated by TLC), the reaction mixture was poured into water and extracted with ethyl acetate (2 × 15 ml). The combined extracts were dried over anhydrous Na_2_SO_4_ and concentrated *in vacuo*. The resulting product was purified by column chromatography on silica gel (Merck, 60–120 mesh, ethyl acetate-hexane, 4:6) to afford the pure product in 88% yield as a white solid. Crystals were grown by slow evaporation from ethanol.

### Refinement

H atoms of the NH_2_ group and water molecules were located in a difference Fourier map and refined with the N—H and O—H distances restrained to 0.90 (1) and 0.85 (1) Å, respectively. H atoms bound to C atoms and the N atoms of the indole rings were placed geometrically and refined using a riding model with d(C—H) = 0.93 Å, d(N—H) = 0.86 Å, and *U*_iso_(H) = 1.2 *U*_eq_(C/N). In the absence of significant anomalous scattering effects, 2271 Friedel pairs were merged as equivalent data.

### Crystal data

**Table:**

C_22_H_12_N_6_O_2_·2H_2_O	*F*_000_ = 888
*M**_r_* = 428.41	*D*_x_ = 1.383 Mg m^−^^3^
Orthorhombic, *P**n**a*2_1_	Mo *K*α radiation λ = 0.71073 Å
Hall symbol: P 2c -2n	Cell parameters from 2386 reflections
*a* = 17.1850 (16) Å	θ = 2.4–28.0º
*b* = 8.9849 (9) Å	µ = 0.10 mm^−^^1^
*c* = 13.3275 (13) Å	*T* = 293 (2) K
*V* = 2057.8 (3) Å^3^	Block, colorless
*Z* = 4	0.25 × 0.24 × 0.20 mm

### Data collection

**Table:**

Bruker SMART APEX CCD diffractometer	2367 reflections with *I* > 2σ(*I*)
Radiation source: fine-focus sealed tube	*R*_int_ = 0.022
Monochromator: graphite	θ_max_ = 28.0º
*T* = 293(2) K	θ_min_ = 2.4º
ω scans	*h* = −22→22
Absorption correction: none	*k* = −11→11
16968 measured reflections	*l* = −17→17
2551 independent reflections	

### Refinement

**Table:**

Refinement on *F*^2^	Secondary atom site location: difference Fourier map
Least-squares matrix: full	Hydrogen site location: inferred from neighbouring sites
*R*[*F*^2^ > 2σ(*F*^2^)] = 0.042	H atoms treated by a mixture of independent and constrained refinement
*wR*(*F*^2^) = 0.108	*w* = 1/[σ^2^(*F*_o_^2^) + (0.0751*P*)^2^ + 0.1068*P*] where *P* = (*F*_o_^2^ + 2*F*_c_^2^)/3
*S* = 1.06	(Δ/σ)_max_ < 0.001
2551 reflections	Δρ_max_ = 0.27 e Å^−^^3^
313 parameters	Δρ_min_ = −0.14 e Å^−^^3^
9 restraints	Extinction correction: none
Primary atom site location: structure-invariant direct methods	

### Special details

**Table:**

Geometry. All e.s.d.'s (except the e.s.d. in the dihedral angle between two l.s. planes) are estimated using the full covariance matrix. The cell e.s.d.'s are taken into account individually in the estimation of e.s.d.'s in distances, angles and torsion angles; correlations between e.s.d.'s in cell parameters are only used when they are defined by crystal symmetry. An approximate (isotropic) treatment of cell e.s.d.'s is used for estimating e.s.d.'s involving l.s. planes.
Refinement. Refinement of *F*^2^ against ALL reflections. The weighted *R*-factor *wR* and goodness of fit *S* are based on *F*^2^, conventional *R*-factors *R* are based on *F*, with *F* set to zero for negative *F*^2^. The threshold expression of *F*^2^ > σ(*F*^2^) is used only for calculating *R*-factors(gt) *etc*. and is not relevant to the choice of reflections for refinement. *R*-factors based on *F*^2^ are statistically about twice as large as those based on *F*, and *R*- factors based on ALL data will be even larger.

### Fractional atomic coordinates and isotropic or equivalent isotropic displacement parameters (Å^2^)

**Table:**

	*x*	*y*	*z*	*U*_iso_*/*U*_eq_	
C1	0.19406 (12)	0.1506 (2)	0.03645 (16)	0.0341 (4)	
C2	0.19661 (11)	0.0810 (2)	0.14549 (15)	0.0320 (4)	
C3	0.11505 (12)	0.1206 (2)	0.19253 (16)	0.0352 (4)	
C4	0.09089 (12)	0.2598 (2)	0.13339 (17)	0.0349 (4)	
C5	0.13624 (13)	0.2738 (2)	0.05081 (16)	0.0354 (4)	
C6	0.16280 (14)	0.0331 (2)	−0.03919 (16)	0.0414 (5)	
C7	0.28704 (15)	0.0835 (3)	−0.08495 (19)	0.0452 (5)	
C8	0.35505 (18)	0.0831 (4)	−0.1392 (2)	0.0614 (7)	
H8A	0.3636	0.0143	−0.1902	0.074*	
C9	0.41025 (19)	0.1889 (4)	−0.1150 (3)	0.0679 (8)	
H9A	0.4570	0.1906	−0.1501	0.082*	
C10	0.39785 (17)	0.2922 (4)	−0.0399 (3)	0.0612 (7)	
H10A	0.4358	0.3629	−0.0256	0.073*	
C11	0.32867 (16)	0.2907 (3)	0.0144 (2)	0.0489 (6)	
H11A	0.3198	0.3607	0.0645	0.059*	
C12	0.27339 (13)	0.1839 (2)	−0.00708 (16)	0.0375 (4)	
C13	0.25886 (12)	0.1685 (2)	0.20696 (15)	0.0340 (4)	
C14	0.29683 (12)	−0.0747 (2)	0.20308 (17)	0.0378 (4)	
C15	0.34057 (15)	−0.2019 (3)	0.2170 (2)	0.0493 (6)	
H15A	0.3882	−0.1992	0.2501	0.059*	
C16	0.31063 (17)	−0.3336 (3)	0.1795 (2)	0.0564 (7)	
H16A	0.3388	−0.4211	0.1878	0.068*	
C17	0.24070 (18)	−0.3388 (3)	0.1305 (2)	0.0581 (7)	
H17A	0.2221	−0.4293	0.1065	0.070*	
C18	0.19709 (16)	−0.2097 (2)	0.1161 (2)	0.0489 (6)	
H18A	0.1497	−0.2129	0.0824	0.059*	
C19	0.22569 (13)	−0.0769 (2)	0.15289 (16)	0.0364 (4)	
C20	0.12114 (15)	0.1500 (3)	0.30204 (19)	0.0449 (5)	
C21	0.05602 (14)	0.0018 (3)	0.1798 (2)	0.0470 (5)	
C22	0.12327 (16)	0.3857 (3)	−0.0210 (2)	0.0506 (6)	
N1	0.22129 (14)	−0.0067 (2)	−0.09965 (16)	0.0486 (5)	
H1	0.2189	−0.0785	−0.1422	0.058*	
N2	0.31446 (11)	0.0717 (2)	0.23268 (15)	0.0413 (4)	
H2	0.3564	0.0962	0.2639	0.050*	
N3	0.12535 (18)	0.1713 (4)	0.38515 (19)	0.0706 (7)	
N4	0.01054 (15)	−0.0884 (3)	0.1731 (3)	0.0727 (8)	
N5	0.03222 (13)	0.3429 (3)	0.16605 (18)	0.0498 (5)	
H5A	0.0025 (15)	0.311 (3)	0.2169 (17)	0.048 (7)*	
H5B	0.011 (2)	0.417 (3)	0.130 (3)	0.070 (10)*	
N6	0.1106 (2)	0.4754 (4)	−0.0785 (3)	0.0916 (11)	
O1	0.09597 (11)	−0.0125 (2)	−0.04096 (16)	0.0567 (5)	
O2	0.25526 (10)	0.29951 (16)	0.22853 (13)	0.0429 (4)	
O3	0.05768 (12)	0.7461 (2)	0.83632 (15)	0.0540 (4)	
H3A	0.073 (3)	0.830 (3)	0.858 (3)	0.107 (15)*	
H3B	0.054 (4)	0.692 (4)	0.888 (2)	0.15 (2)*	
O4	0.0238 (2)	0.3832 (4)	0.5501 (3)	0.0938 (9)	
H4A	0.020 (4)	0.322 (6)	0.599 (4)	0.16 (3)*	
H4B	0.066 (2)	0.360 (6)	0.521 (4)	0.13 (2)*	

### Atomic displacement parameters (Å^2^)

**Table:**

	*U*^11^	*U*^22^	*U*^33^	*U*^12^	*U*^13^	*U*^23^
C1	0.0439 (10)	0.0289 (9)	0.0294 (9)	0.0008 (8)	−0.0003 (8)	−0.0036 (7)
C2	0.0387 (9)	0.0268 (9)	0.0304 (9)	−0.0010 (7)	−0.0022 (8)	−0.0033 (7)
C3	0.0384 (9)	0.0334 (9)	0.0338 (10)	−0.0009 (8)	0.0006 (8)	0.0015 (8)
C4	0.0404 (10)	0.0319 (9)	0.0325 (9)	−0.0002 (7)	−0.0024 (8)	−0.0019 (8)
C5	0.0428 (10)	0.0329 (10)	0.0305 (9)	0.0024 (8)	−0.0017 (8)	−0.0015 (8)
C6	0.0565 (13)	0.0354 (10)	0.0324 (10)	0.0013 (9)	−0.0081 (10)	−0.0082 (8)
C7	0.0582 (13)	0.0408 (11)	0.0367 (11)	0.0131 (10)	0.0031 (10)	−0.0020 (9)
C8	0.0700 (18)	0.0639 (17)	0.0503 (15)	0.0182 (13)	0.0193 (14)	−0.0015 (13)
C9	0.0570 (16)	0.083 (2)	0.0642 (18)	0.0135 (15)	0.0240 (14)	0.0145 (17)
C10	0.0539 (15)	0.0616 (16)	0.0682 (19)	−0.0052 (12)	0.0088 (14)	0.0140 (14)
C11	0.0552 (14)	0.0411 (12)	0.0504 (14)	−0.0037 (10)	0.0070 (11)	−0.0017 (10)
C12	0.0449 (11)	0.0353 (10)	0.0321 (10)	0.0060 (8)	0.0030 (9)	0.0003 (8)
C13	0.0407 (9)	0.0341 (9)	0.0271 (9)	−0.0040 (8)	0.0010 (8)	−0.0036 (7)
C14	0.0443 (10)	0.0351 (10)	0.0340 (10)	0.0029 (8)	0.0020 (9)	−0.0012 (8)
C15	0.0517 (13)	0.0486 (13)	0.0476 (13)	0.0143 (10)	0.0002 (11)	0.0010 (11)
C16	0.0687 (16)	0.0374 (11)	0.0632 (16)	0.0167 (11)	0.0081 (13)	0.0036 (11)
C17	0.0764 (18)	0.0286 (11)	0.0692 (18)	0.0006 (11)	0.0009 (14)	−0.0073 (11)
C18	0.0586 (14)	0.0320 (10)	0.0563 (14)	−0.0015 (9)	−0.0054 (12)	−0.0061 (10)
C19	0.0443 (10)	0.0300 (9)	0.0351 (10)	0.0021 (8)	0.0006 (9)	−0.0004 (8)
C20	0.0473 (12)	0.0483 (12)	0.0392 (13)	0.0044 (10)	0.0049 (10)	0.0056 (10)
C21	0.0447 (11)	0.0433 (12)	0.0530 (14)	−0.0024 (10)	−0.0003 (11)	0.0055 (10)
C22	0.0640 (15)	0.0478 (12)	0.0400 (12)	0.0167 (11)	0.0151 (11)	0.0054 (10)
N1	0.0696 (13)	0.0394 (10)	0.0368 (10)	0.0049 (9)	−0.0004 (10)	−0.0135 (8)
N2	0.0418 (9)	0.0414 (9)	0.0406 (10)	0.0006 (7)	−0.0076 (8)	−0.0047 (8)
N3	0.0812 (18)	0.0944 (19)	0.0363 (13)	0.0063 (14)	0.0041 (11)	0.0020 (12)
N4	0.0595 (13)	0.0613 (14)	0.097 (2)	−0.0217 (12)	−0.0013 (14)	0.0057 (15)
N5	0.0509 (11)	0.0526 (12)	0.0458 (11)	0.0136 (9)	0.0108 (10)	0.0073 (9)
N6	0.121 (3)	0.084 (2)	0.0696 (18)	0.0470 (19)	0.0373 (18)	0.0380 (16)
O1	0.0578 (11)	0.0560 (10)	0.0563 (11)	−0.0089 (8)	−0.0123 (9)	−0.0163 (9)
O2	0.0552 (9)	0.0330 (7)	0.0404 (8)	−0.0026 (6)	−0.0050 (7)	−0.0110 (6)
O3	0.0606 (11)	0.0517 (9)	0.0498 (10)	0.0029 (9)	−0.0094 (9)	−0.0077 (8)
O4	0.093 (2)	0.091 (2)	0.097 (2)	0.0319 (15)	−0.0022 (16)	0.0075 (18)

### Geometric parameters (Å, °)

**Table:**

C1—C5	1.500 (3)	C11—H11A	0.930
C1—C12	1.511 (3)	C13—O2	1.213 (3)
C1—C6	1.556 (3)	C13—N2	1.337 (3)
C1—C2	1.583 (3)	C14—C15	1.380 (3)
C2—C19	1.508 (3)	C14—C19	1.394 (3)
C2—C13	1.560 (3)	C14—N2	1.406 (3)
C2—C3	1.576 (3)	C15—C16	1.384 (4)
C3—C21	1.482 (3)	C15—H15A	0.930
C3—C20	1.487 (3)	C16—C17	1.369 (4)
C3—C4	1.535 (3)	C16—H16A	0.930
C4—N5	1.328 (3)	C17—C18	1.394 (4)
C4—C5	1.354 (3)	C17—H17A	0.930
C5—C22	1.406 (3)	C18—C19	1.380 (3)
C6—O1	1.220 (3)	C18—H18A	0.930
C6—N1	1.337 (3)	C20—N3	1.126 (4)
C7—C8	1.374 (4)	C21—N4	1.130 (3)
C7—C12	1.395 (3)	C22—N6	1.133 (4)
C7—N1	1.404 (3)	N1—H1	0.860
C8—C9	1.381 (5)	N2—H2	0.860
C8—H8A	0.930	N5—H5A	0.90 (1)
C9—C10	1.382 (5)	N5—H5B	0.90 (1)
C9—H9A	0.930	O3—H3A	0.85 (1)
C10—C11	1.392 (4)	O3—H3B	0.85 (1)
C10—H10A	0.930	O4—H4A	0.85 (1)
C11—C12	1.380 (3)	O4—H4B	0.85 (1)
			
C5—C1—C12	120.04 (17)	C12—C11—H11A	120.4
C5—C1—C6	110.78 (17)	C10—C11—H11A	120.4
C12—C1—C6	101.37 (17)	C11—C12—C7	119.2 (2)
C5—C1—C2	101.12 (16)	C11—C12—C1	132.7 (2)
C12—C1—C2	113.93 (17)	C7—C12—C1	108.0 (2)
C6—C1—C2	109.64 (16)	O2—C13—N2	127.39 (19)
C19—C2—C13	102.28 (17)	O2—C13—C2	125.34 (19)
C19—C2—C3	118.76 (17)	N2—C13—C2	107.25 (16)
C13—C2—C3	106.69 (16)	C15—C14—C19	122.0 (2)
C19—C2—C1	116.18 (17)	C15—C14—N2	128.3 (2)
C13—C2—C1	107.57 (15)	C19—C14—N2	109.72 (18)
C3—C2—C1	104.56 (15)	C14—C15—C16	117.2 (2)
C21—C3—C20	106.75 (19)	C14—C15—H15A	121.4
C21—C3—C4	110.04 (18)	C16—C15—H15A	121.4
C20—C3—C4	112.24 (19)	C17—C16—C15	121.9 (2)
C21—C3—C2	113.62 (17)	C17—C16—H16A	119.1
C20—C3—C2	111.58 (18)	C15—C16—H16A	119.1
C4—C3—C2	102.72 (16)	C16—C17—C18	120.6 (2)
N5—C4—C5	130.6 (2)	C16—C17—H17A	119.7
N5—C4—C3	119.7 (2)	C18—C17—H17A	119.7
C5—C4—C3	109.72 (18)	C19—C18—C17	118.6 (3)
C4—C5—C22	121.9 (2)	C19—C18—H18A	120.7
C4—C5—C1	114.59 (18)	C17—C18—H18A	120.7
C22—C5—C1	123.10 (19)	C18—C19—C14	119.7 (2)
O1—C6—N1	127.3 (2)	C18—C19—C2	132.2 (2)
O1—C6—C1	124.5 (2)	C14—C19—C2	107.96 (18)
N1—C6—C1	108.2 (2)	N3—C20—C3	179.4 (3)
C8—C7—C12	122.4 (3)	N4—C21—C3	178.0 (3)
C8—C7—N1	127.5 (2)	N6—C22—C5	178.1 (3)
C12—C7—N1	110.0 (2)	C6—N1—C7	111.51 (19)
C7—C8—C9	117.3 (3)	C6—N1—H1	124.2
C7—C8—H8A	121.3	C7—N1—H1	124.2
C9—C8—H8A	121.3	C13—N2—C14	112.51 (18)
C8—C9—C10	121.7 (3)	C13—N2—H2	123.7
C8—C9—H9A	119.1	C14—N2—H2	123.7
C10—C9—H9A	119.1	C4—N5—H5A	119.9 (19)
C9—C10—C11	120.1 (3)	C4—N5—H5B	123 (3)
C9—C10—H10A	119.9	H5A—N5—H5B	115 (3)
C11—C10—H10A	119.9	H3A—O3—H3B	105.1 (17)
C12—C11—C10	119.1 (2)	H4A—O4—H4B	104.8 (17)
			
C5—C1—C2—C19	158.77 (17)	C9—C10—C11—C12	0.7 (4)
C12—C1—C2—C19	−71.0 (2)	C10—C11—C12—C7	−2.2 (4)
C6—C1—C2—C19	41.8 (2)	C10—C11—C12—C1	179.7 (2)
C5—C1—C2—C13	−87.37 (18)	C8—C7—C12—C11	2.5 (4)
C12—C1—C2—C13	42.8 (2)	N1—C7—C12—C11	−175.0 (2)
C6—C1—C2—C13	155.61 (17)	C8—C7—C12—C1	−179.0 (2)
C5—C1—C2—C3	25.79 (18)	N1—C7—C12—C1	3.5 (3)
C12—C1—C2—C3	155.99 (17)	C5—C1—C12—C11	48.3 (4)
C6—C1—C2—C3	−91.22 (18)	C6—C1—C12—C11	170.6 (2)
C19—C2—C3—C21	−37.6 (3)	C2—C1—C12—C11	−71.8 (3)
C13—C2—C3—C21	−152.32 (18)	C5—C1—C12—C7	−130.0 (2)
C1—C2—C3—C21	93.88 (19)	C6—C1—C12—C7	−7.7 (2)
C19—C2—C3—C20	83.1 (2)	C2—C1—C12—C7	110.0 (2)
C13—C2—C3—C20	−31.6 (2)	C19—C2—C13—O2	−173.2 (2)
C1—C2—C3—C20	−145.38 (18)	C3—C2—C13—O2	−47.8 (3)
C19—C2—C3—C4	−156.44 (18)	C1—C2—C13—O2	63.9 (3)
C13—C2—C3—C4	88.85 (18)	C19—C2—C13—N2	5.2 (2)
C1—C2—C3—C4	−24.95 (19)	C3—C2—C13—N2	130.60 (18)
C21—C3—C4—N5	72.4 (3)	C1—C2—C13—N2	−117.67 (18)
C20—C3—C4—N5	−46.3 (3)	C19—C14—C15—C16	−0.5 (4)
C2—C3—C4—N5	−166.3 (2)	N2—C14—C15—C16	−179.2 (2)
C21—C3—C4—C5	−106.6 (2)	C14—C15—C16—C17	0.1 (4)
C20—C3—C4—C5	134.7 (2)	C15—C16—C17—C18	0.4 (5)
C2—C3—C4—C5	14.7 (2)	C16—C17—C18—C19	−0.4 (5)
N5—C4—C5—C22	−3.5 (4)	C17—C18—C19—C14	0.0 (4)
C3—C4—C5—C22	175.3 (2)	C17—C18—C19—C2	175.9 (3)
N5—C4—C5—C1	−176.4 (2)	C15—C14—C19—C18	0.4 (4)
C3—C4—C5—C1	2.4 (3)	N2—C14—C19—C18	179.4 (2)
C12—C1—C5—C4	−144.5 (2)	C15—C14—C19—C2	−176.4 (2)
C6—C1—C5—C4	97.9 (2)	N2—C14—C19—C2	2.6 (3)
C2—C1—C5—C4	−18.3 (2)	C13—C2—C19—C18	179.1 (3)
C12—C1—C5—C22	42.7 (3)	C3—C2—C19—C18	62.1 (4)
C6—C1—C5—C22	−74.9 (3)	C1—C2—C19—C18	−64.0 (3)
C2—C1—C5—C22	168.9 (2)	C13—C2—C19—C14	−4.6 (2)
C5—C1—C6—O1	−41.8 (3)	C3—C2—C19—C14	−121.6 (2)
C12—C1—C6—O1	−170.3 (2)	C1—C2—C19—C14	112.2 (2)
C2—C1—C6—O1	68.9 (3)	O1—C6—N1—C7	171.7 (2)
C5—C1—C6—N1	138.14 (19)	C1—C6—N1—C7	−8.3 (3)
C12—C1—C6—N1	9.6 (2)	C8—C7—N1—C6	−174.1 (3)
C2—C1—C6—N1	−111.1 (2)	C12—C7—N1—C6	3.2 (3)
C12—C7—C8—C9	−1.1 (4)	O2—C13—N2—C14	174.4 (2)
N1—C7—C8—C9	176.0 (3)	C2—C13—N2—C14	−4.0 (2)
C7—C8—C9—C10	−0.5 (5)	C15—C14—N2—C13	179.9 (2)
C8—C9—C10—C11	0.7 (5)	C19—C14—N2—C13	1.0 (3)

### Hydrogen-bond geometry (Å, °)

**Table:**

*D*—H···*A*	*D*—H	H···*A*	*D*···*A*	*D*—H···*A*
N1—H1···O2^i^	0.86	2.09	2.905 (2)	158
N2—H2···O3^i^	0.86	2.22	3.032 (3)	157
O3—H3A···O1^ii^	0.85 (1)	2.00 (2)	2.795 (3)	157 (4)
O3—H3B···N6^iii^	0.85 (1)	2.22 (5)	2.834 (3)	129 (5)
O3—H3B···O4^iv^	0.85 (1)	2.62 (3)	3.381 (4)	149 (5)
O4—H4A···N4^v^	0.85 (1)	2.38 (3)	3.171 (5)	155 (7)
O4—H4B···N3	0.85 (1)	2.68 (4)	3.392 (4)	142 (6)
N5—H5A···O3^vi^	0.90 (1)	1.97 (1)	2.860 (3)	175 (3)
N5—H5B···O4^vi^	0.90 (1)	2.17 (1)	3.061 (4)	171 (3)

Symmetry codes: (i) −*x*+1/2, *y*−1/2, *z*−1/2; (ii) *x*, *y*+1, *z*+1; (iii) *x*, *y*, *z*+1; (iv) −*x*, −*y*+1, *z*+1/2; (v) −*x*, −*y*, *z*+1/2; (vi) −*x*, −*y*+1, *z*−1/2.
